# Efficient and Highly Accurate Diagnosis of Malignant Hematological Diseases Based on Whole-Slide Images Using Deep Learning

**DOI:** 10.3389/fonc.2022.879308

**Published:** 2022-06-10

**Authors:** Chong Wang, Xiu-Li Wei, Chen-Xi Li, Yang-Zhen Wang, Yang Wu, Yan-Xiang Niu, Chen Zhang, Yi Yu

**Affiliations:** ^1^ School of Biological Science and Medical Engineering, Beihang University, Beijing, China; ^2^ School of Medical Engineering, Xinxiang Medical University, Xinxiang, China; ^3^ Department of Neurobiology, School of Basic Medical Sciences, Beijing Key Laboratory of Neural Regeneration and Repair, Advanced Innovation Center for Human Brain Protection, Capital Medical University, Beijing, China; ^4^ Department of Hematology, Xinxiang First People's Hospital, Xinxiang, China; ^5^ School of Life Sciences, Tsinghua University, Beijing, China; ^6^ Henan Province Neural Sensing and Control Engineering Technology Research Center, Xinxiang, China; ^7^ Chinese Institute for Brain Research, Beijing, China

**Keywords:** hematological malignancies, deep learning, digital pathology, weakly supervised, hematopathology

## Abstract

Hematopoietic disorders are serious diseases that threaten human health, and the diagnosis of these diseases is essential for treatment. However, traditional diagnosis methods rely on manual operation, which is time consuming and laborious, and examining entire slide is challenging. In this study, we developed a weakly supervised deep learning method for diagnosing malignant hematological diseases requiring only slide-level labels. The method improves efficiency by converting whole-slide image (WSI) patches into low-dimensional feature representations. Then the patch-level features of each WSI are aggregated into slide-level representations by an attention-based network. The model provides final diagnostic predictions based on these slide-level representations. By applying the proposed model to our collection of bone marrow WSIs at different magnifications, we found that an area under the receiver operating characteristic curve of 0.966 on an independent test set can be obtained at 10× magnification. Moreover, the performance on microscopy images can achieve an average accuracy of 94.2% on two publicly available datasets. In conclusion, we have developed a novel method that can achieve fast and accurate diagnosis in different scenarios of hematological disorders.

## 1 Introduction

Hematopoietic disorders are complex diseases, and their early diagnosis is critical for proposing correct treatments (1–3). The diagnosis, prognosis, and follow-up of most hematological diseases, especially hematologic malignancies [e.g., acute myeloid leukemia (AML) and acute lymphoid leukemia (ALL)] are strongly dependent on the manual examination of the bone marrow (4, 5). In the traditional analysis of bone marrow smears, a hematologist first selects the regions of interest (ROIs) with the appropriate distribution of cellular trails, usually at the body end of the smear, and then performs a morphological analysis of hundreds of cells in the ROIs (6, 7). Hence, in this manner, the examiner’s effort is considerable, and the accuracy is strongly dependent on the expertise level of the examiner. The morphological differences in the bone marrow cell developmental stages during diagnosis are small and prone to inter-observer variability, with studies showing inter-observer kappa averages ranging from 0.352 to 0.630 (8, 9).

In recent years, the development of digital imaging technology has facilitated the use of whole-slide images (WSIs) for tumor diagnosis (10, 11), tumor origin (12, 13), prognostic analysis (14, 15), and other digital pathology developments, which have improved the efficiency and accuracy of clinical diagnosis. For digital pathology, glass slides are scanned to generate files that typically have several gigapixels (20× magnification), and slide-level labels are only relevant to tiny regions in the WSI (16, 17). The peculiarity of WSI has led most efforts in digital pathology to rely on applying supervised learning to classify small patches, which requires extensive annotation at the pixel level (18, 19). Recent studies shown that deep learning methods based on a variant of multiple- instance learning (MIL) for analyzing WSI in a weakly supervised environment exhibit excellent performance (20–22). The MIL directly utilizes slide-level labels, assigns patches to the same labels as slides, and predicts cancer if the k highest scoring patches are predicted to be cancer (23). However, these methods require thousands of slides for training to obtain a performance comparable to those of fully supervised methods, and clinical data collection on such a huge scale is difficult, especially for some rare diagnoses (17). Recently, Lu et al. proposed an attention-based MIL to predict the origin of cancer and achieved a high-performance accuracy of 0.96 using only slide-level labels (12). The MIL-based method’s performance indicated that weakly supervised deep learning methods can be competent for medical diagnosis, significantly reducing the difficulty of data collection.

The development of artificial intelligence provides opportunities for the intelligent diagnosis of hematological diseases, and studies attempting to diagnose leukemia through a direct analysis of microscopic images have been reported. Huang et al. achieved the classification of AML, ALL, and chronic myeloid leukemia (CML) (24) using DenseNet121, having an accuracy of 95.3%. Shafique et al. and Rehman et al. used convolutional neural networks (CNNs) to realize the subtype classification of ALL, achieving accuracies of 97.78% and 96.06% (25, 26). However, these methods lack interpretability studies and use limited imaging. By creating large-scale cell annotation datasets, studies have achieved expert-level nucleated cell differential counting (NDC) of bone marrow micrographs or single-cell images using CNNs (27–30). However, these methods still require manual involvement to obtain the ROIs and locate cellular trails. Recently, Wang et al. achieved the fully automated analysis of bone marrow smears through NDC using WSIs, which automatically selects ROIs at low magnifications, followed by cell counting under a 40× oil microscope, achieving a recall performance of 0.90 (31). However, this method requires thousands of cell-level labels, data acquisition is difficult, and the bone marrow cell developmental diversity leads to inter-observer variability, which affects the quality of annotation (32, 33).

In this study, we developed a weakly supervised method that can be applied to hematopoietic disorders, especially hematological malignancies. To the authors’ knowledge, this is the first report using only slide-level labels to diagnose hematological diseases. Our method achieves high performance using only slide-level labels, and the proposed model is data-efficient and interpretable. It aims to address the drawback of the heavy reliance on the manual detection of hematological malignancies.

## 2 Materials And Methods

### 2.1 WSI Dataset

The Ethics Committee ethically approved this study of Xinxiang Medical University (2019S026). All bone marrow aspirate smears used were historical samples, which were identified, photographed, and preserved by experts before inclusion in the study. Due to its retrospective design, informed patient consent was waived. For the in-house dataset, we collected bone marrow-stained slides of 129 patients from the First People’s Hospital of Xinxiang City for 5-fold cross-validation and 30 patients from the Third Affiliated Hospital of Xinxiang Medical University as an independent test set. Fifty-five of the slides were AML, 20 were CML, 31 were ALL, 29 were chronic lymphoid leukemia (CLL), and 24 were multiple myeloma (MM) (**Table 1**). All slides were imaged using an Austar43 scanner (AiMco, Xiamen/China) in 10×, 40×, and oil-immersion 100× objective lens scans. Slides were collected from a selection of in-house case files from 2015 to 2020.

**Table 1:** Dataset description.

	AML	CML	ALL	CLL	MM
In-house dataset	45	16	25	23	20
External dataset	10	4	6	6	4
Total	55	20	31	29	24

### 2.2 Public Dataset

We used micrographs from three publicly available datasets, ALL-IDB (34), SN-AM(ALL), and SN-AM(MM) (35), to test the performance of our model. ALL-IDB is a public dataset of ALL patients’ peripheral blood, containing ALL-IDB1 and ALL-IDB2, where ALL-IDB2 contains only single-cell images. Thus, we only analyzed ALL-IDB1, which was captured using the Canon PowerShot G5 with a resolution of 2592 × 1944 and magnification range from 300 to 500 and included 49 images of ALL and 59 images of healthy individuals (36). The SN-AM(ALL) and SN-AM(MM) included bone marrow aspirate smears prepared with Jenner–Giemsa stain from patients diagnosed with ALL and MM and contained 30 images for each dataset. The images were captured at 100× magnification with a resolution of 2560 × 1920. These datasets were also available for public download from The Cancer Imaging Archive (37).

### 2.3 Data Preprocessing

#### 2.3.1 WSI

WSIs are huge, especially at 100× magnification (slide dimensions in pixels is ~7,000,000 × ~10,000,000), making them difficult to be analyzed. The ROI [as recommended by the International Council for Standardization in Hematology guidelines (6)] and other regions (containing dense cell distribution and non-cells view) were manually randomly selected in the training dataset for 5-fold cross-validation to improve efficiency. The manually selected region accounts for 5.15% of each WSI with an average size of 220000 × 98304 pixels, 22.81 mm^2^ at 100× magnification. The selected regions were used in the training phase, and the entire WSI was applied to validate and test to ensure the results reliability. The background in each digitized slide was subsequently filtered out using the Otsu (38) algorithm to reduce unnecessary computations at a 16× downsampled resolution, and the foreground region was cropped into 256 × 256 patches. After background removal, an average of 0.33, 6.65, and 6.15 million patches per WSI were included in the training, validation, and test datasets.

#### 2.3.2 Microscopy Images

We treated each micrograph as a separate individual because the public datasets do not have detailed annotations for each image. All images were subsequently downsampled to a magnification of 10× and copied into 256 × 256 patches. The number of extracted patches per set ranged from 30 to 70.

### 2.4 Network Structure and Training

#### 2.4.1 Model Architecture

For the WSI and microscopic images, after preprocessing, the ResNet50 (39) pretrained on ImageNet was utilized to convert each 256 × 256 patch into a 1024-dimensional feature vector. The computed low-dimensional features were then fed into a weakly supervised deep learning framework for training, which is based on the clustering-constrained-attention multiple-instance learning (CLAM) framework (17). The proposed framework had *N* parallel attention branches for predicting *N* attention scores for each patch, corresponding to each category of the classification task. By assigning different category attention scores to each patch, the model can explicitly learn which patches were positive features of a particular category and then summarize each category’s unique slide-level representations. Finally, each category of the slide representation was examined by a classification layer to obtain the final predictions of the WSI. Specifically, the two fully connected layers Fc1 and Fc2 with the parameter of 1024, 512 neurons converted each patch feature vector into a 512-dimensional vector and each Fc layer followed by rectified linear unit activation. Fc2 was followed by an attention network consisting of several fully connected layers, with the first two fully connected layers Attention-Fc1 and Attention-Fc2 with weight parameters *W_attn_*_1_ *∈* ℝ^384×512^ and *W_attn_*_2_ *∈* ℝ^384×512^. The attention network then splits into *N* parallel attention branches *P_a_*_,1_,…,*P_a,n_ ∈* ℝ^1×384^ to compute patch feature class attention score. Each patch attention score *s_i,k_* was calculated:


(1) $$s_{i,k}=\frac{\text{exp}\{P_{a,i}(\text{tanh}(W_{attn1}{\text{h}}_{k})⊙\text{sigmoid(}W_{attn2}{\text{h}}_{k})\}}{\sum_{j=1}^{N}\text{exp}\{P_{a,i}(\text{tanh}(W_{attn1}{\text{h}}_{k})⊙\text{sigmoid(}W_{attn2}{\text{h}}_{k})\}}$$

where **h** *_k_* is the *k*th patch feature, *i* is the corresponding class, ⊙ is the element-wise product, and the bias parameters are excluded from the equation for simplicity. And *N* parallel independent classifiers (*W_c_*_,1_,…,*W_c,n_ ∈* ℝ^1×512^) were built to score each class-specific slide-level representation. The slide-level score for the *i*th class was calculated:


(2) $$\begin{array}{c} s_{slide,i}=W_{c,i}{\text{h}}_{slide,i}\\ =W_{c,i}(\sum_{k=1}^{K}a_{i,k}{\text{h}}_{k}) \end{array}$$

where *K* the number of patches in a WSI, and ***h*** *_slide_*,*_i_* is the slide-level representation. After each attention backbone layer, we used dropout (P=0.3) for regularization. Then, we predicted the slide-level scores for each class using the softmax function.

#### 2.4.2 Instance-Level Clustering

Similar with CLAM (17), instance-level clustering was used to further learn the class features. The instance-level clustering layer was placed after Fc2. The attention network clusters the positive and negative features of each class by optimizing a subset of the number of *B* patches with the most and least attention. The smooth SVM loss function (40) was used as the loss function for the instance-level clustering task.

#### 2.4.3 Training Details

The model uses a batch size of one during training. The number of patches *B* sampled from the in-the-class branch is different in each magnification: 8 in the 10× magnification, 32 in the 40× magnification, and 128 in the 100× magnification. The Adam optimization algorithm minimizes the loss functions, and the learning rate is 0.0002. All models have 200 trained epochs if the early stopping criterion (the validation loss has not decreased over 20 epochs) is not met.

### 2.5 Model Interpretability

For deep learning classification tasks, it is important to intuitively explain the reasons for predicting categories. We performed this by tiling the foreground regions of the WSI into 256 × 256 patches and calculating the attention scores for each patch. Subsequently, they were scaled *via* normalization to between 0 and 1.0 (the larger the score, the higher the model attention), and an overlap of 50% was used in tiling the patches for a more fine-grained presentation of the results.

### 2.6 Computational Hardware and Software

All tasks were performed on a workstation with Nvidia RTX 3090 and Intel Xeon CPUs. All codes were implemented based on Python 3.7, mainly using PyTorch, for deep learning model training and OpenSlide, Pillow, OpenCV, and CLAM for WSI analysis.

## 3 Results

### 3.1 Weakly Supervised Deep Learning Method for the Automatic Analysis of Hematological Malignancies

**Figure 1:**
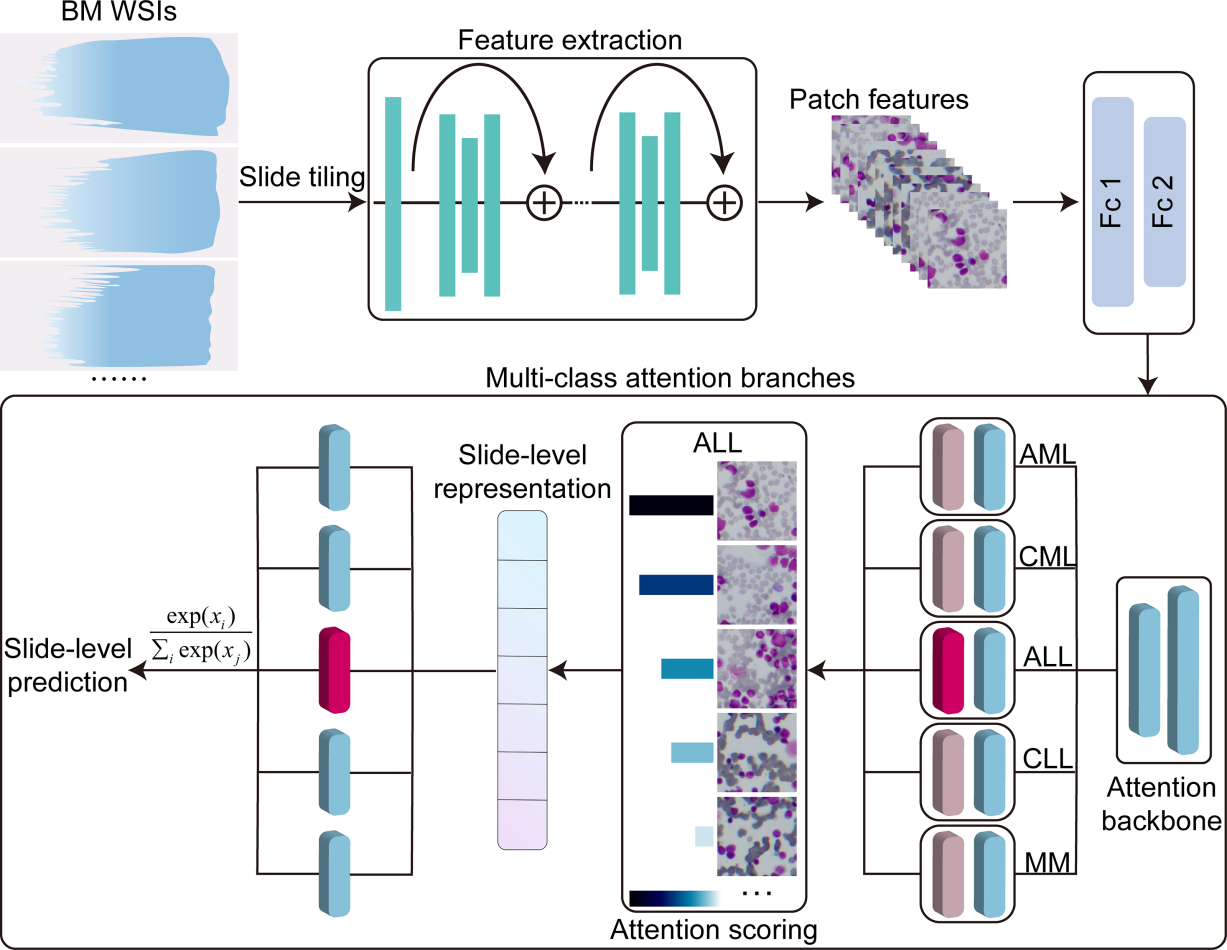
Overview of the model architecture. Bone marrow WSIs were divided into image patches, and then the patches were encoded by a pretrained ResNet50-based CNN fixed into a low-dimensional feature vectors in the training and inference. Multi-class attention branches assign an attention score to each patch in the WSI based on its importance to the slide-level diagnosis and weigh the importance of the patches by the attention score to aggregate the patch features into a slide level representation. Based on these slide-level representations, the model marked the final diagnostic predictions. Fc1, Fc2, fully connected layers; AML, acute myeloid leukemia; ALL, acute lymphoid leukemia; CML, chronic myeloid leukemia; CLL, chronic lymphoid leukemia; MM, multiple myeloma.

Our study aims to develop a weakly supervised deep learning framework using only slide-level label for the automated analysis of bone marrow smears. We collected bone marrow smears from 159 patients (including 30 samples from other hospitals as an independent test set). These sample include five common malignant hematological diseases: AML, ALL, CML, CLL, MM. The computational strategy is summarized in **Figure 1**. First, the model reduced the dimensionality of the WSI patch image using a pretrained feature extraction network. Then, the low-dimensional features were fed into an attention network that contains five parallel attention branches that together compute unique slide-level representations of different hematological malignancies. The representation of each category was determined by the network’s consideration of the regions as strong positive evidence for a particular category in the diagnostic task. We tested the performance of our method on two publicly datasets and an independent test set. The results showed our model obtained a high performance (area under the curve (AUC) >0.95), indicating that it can be effectively applied to solve hematological diagnostic using only the patients’ slide-level labels.

### 3.2 Magnification-Dependent Cross-Validated Model Performance

**Figure 2:**
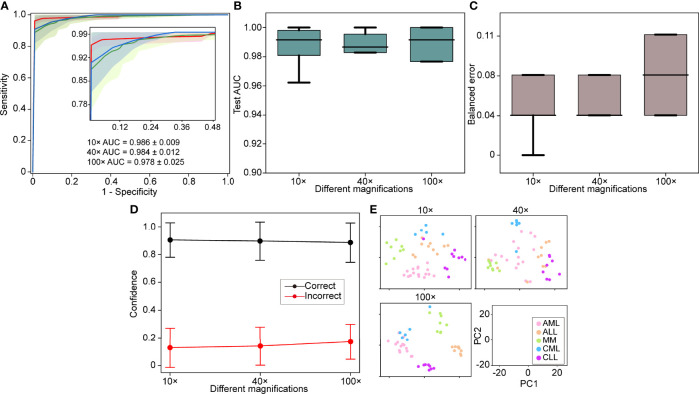
Different magnification performance and comparative analysis. **(A)** 5-fold mean test macro-averaged AUC ± s.d. of our model using 10×, 40×, and 100× objectives. The confidence band shows ±1 s.d. for the ROC. **(B)** Cross-validation test AUCs. **(C)** Cross-validation test balanced error. **(D)** Average confidence (± 1 s.d.) of correctly and incorrectly classified WSIs of the model. **(E)** Slide-level feature space for a single cross-validated fold using PCA in the validation and test sets. PC, principal component.

The 5-fold Monte Carlo cross-validation was used to evaluate our model’s performance at different magnifications (10×, 40×, and 100×). We used the images acquired with different objectives for the magnification performance comparison rather than downsampling the images at high magnification objectives. We randomly divided each category into a training set (60% of cases), a validation set (20% of cases), and a test set (20%) for each cross-validation fold. A manually selected local region was used for training, and the full WSIs were used for validation and testing. On our in-house dataset, the model achieved a 5-fold macro-averaged one-versus-rest mean test AUC ± s.d. of 0.979 ± 0.015 for the five-class hematological malignancy subtypes of AML, ALL, CML, CLL, and MM at 10× magnification (**Figure 2A**), with an average classification accuracy of 90%.

A high magnification means a great image resolution, which also requires great computational resources. In particular, the WSI of a 100× oil lens, which is commonly used for bone marrow slide analysis, is difficult to obtain due to the focus and imaging time. In light of these limitations, the effect of different magnifications on the performance was investigated. The 10× AUC ± s.d was 0.986 ± 0.009, the 40× AUC ± s.d was 0.984 ± 0.012, and the 100× AUC ± s.d was 0.978 ± 0.025. The comparative analysis results show that 10× magnification works better than the others with the same training, validation, and test sets (**Figures 2B** **–D**). We also used a 512-dimensional feature representation per slide for disease prediction visualized after reduction to a two-dimensional space *via* PCA and observed that the learned feature space was clearly separable (**Figure 2E**). Our results showed that excellent performance can be achieved using only 10× magnification, increase imaging efficiency and save computational resources.

### 3.3 Adaptability to Independent Test Cohorts

**Figure 3:**
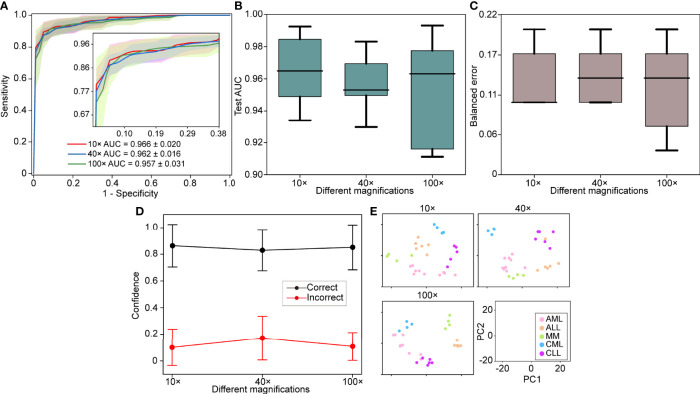
Independent test set performance. **(A)** 5-fold mean test macro-averaged AUC ± s.d. of our model using 10×, 40×, and 100× objectives on the independent test set. The confidence band shows ±1 s.d. for the ROC. **(B)** Cross-validation test AUCs. **(C)** Cross-validation test balanced error. **(D)** Average confidence ( ± 1 s.d.) of correctly and incorrectly classified WSIs of the model. **(E)** Slide-level feature space for a single cross-validated fold using PCA in the validation and test sets.

WSIs may greatly vary due to slide production and staining caused by institutional differences. Therefore, the model should be robust to other hospitals. The 30 slides were collected as an independent test set to evaluate the generalization performance of the model. The independent test set was tested on each of the five models obtained at different magnifications using cross-validation, and we used the average performance of all modes to avoid variances of different models. We found that the performance remains excellent for the independent test set (AUC >0.95), with the best performance at 10× magnification with a macro-averaged AUC ± s.d of 0.966 ± 0.020 as compared to AUC ± s.d of 0.962 ± 0.016 (40×) and 0.957 ± 0.031 (100×) (**Figure 3A**). The independent test set and cross-validation showed the same results, with 10× magnification performing better than the other magnifications (**Figures 3B** **–E**). These results indicated that the robustness of our model to bone marrow smears from different hospitals.

### 3.4 Interpretability of the Results

**Figure 4:**
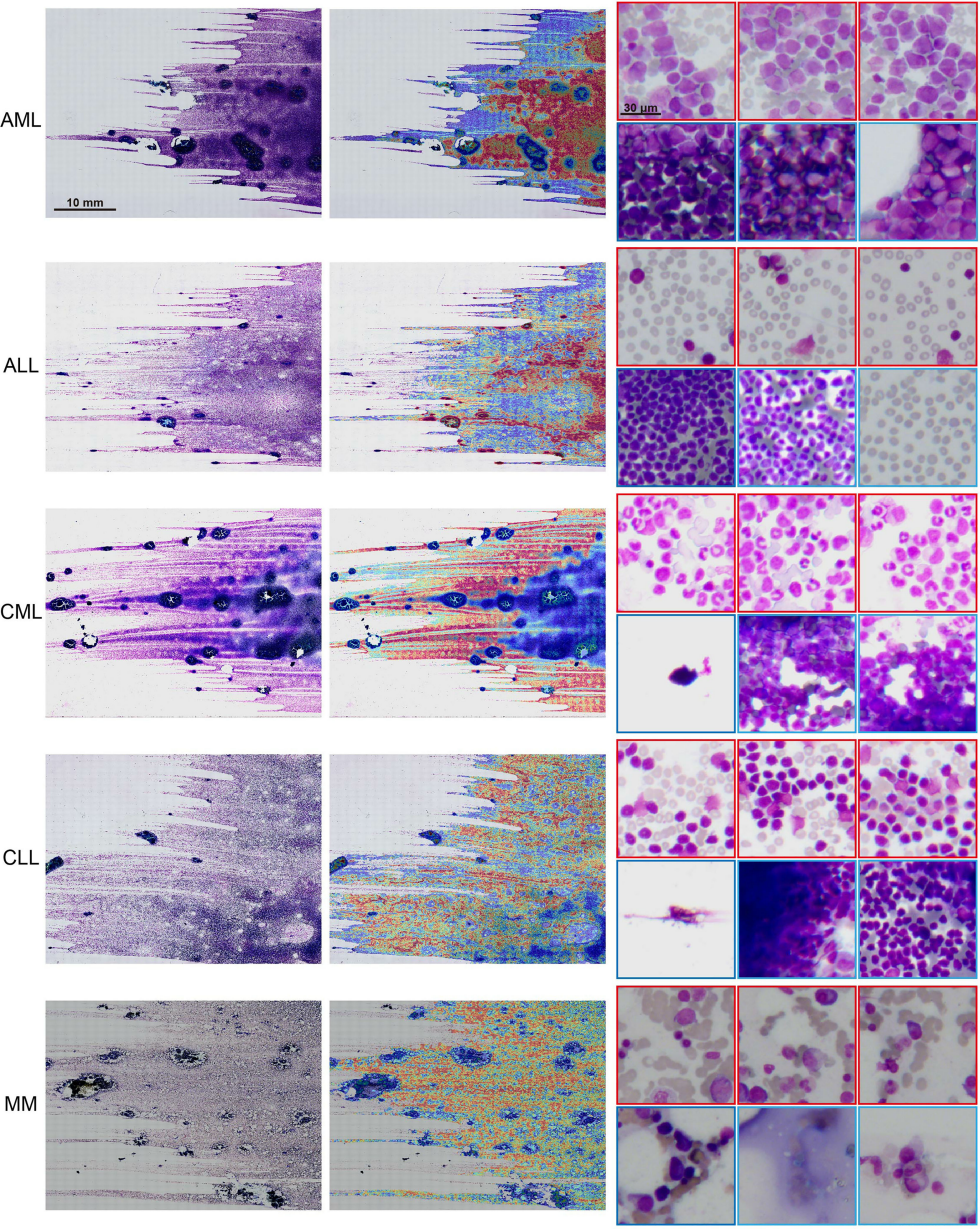
Interpretability and visualization at 10× magnification. Raw WSIs of representative slides of different kinds of malignant blood tumors (left), the generated attention heatmap (middle). The region of strongest attention (red border) usually focuses on the region of interest (ROI) with distributed blast cell tracks, whereas the region of low attention (blue border) includes images with dense cell distribution, no cells, and other kinds of cells (right).

The model interpretability can verify that the predictive foundation of deep learning is consistent with the concerns of pathologists and can also be used to analyze erroneous results. We used the regional attention scores of the model prediction categories mapped to the corresponding spatial locations *via* normalization and used overlapping patches and average scores to create fine-grained attention heatmaps to explain the model classification results. Despite the absence of pixel-level labeling of the ROIs, the model still observed that areas with a uniform distribution of mature erythrocyte cells and a clear leukocyte structure were the best areas to determine the type of disease (**Figure 4**). The areas of high concern were the same as those where hematologists determine diseases based on cell types. For example, there was a high concern for patches with myeloblasts, and areas with only other cells showed a low score for AML.

### 3.5 Generalization to Public Microscopy Images

**Figure 5:**
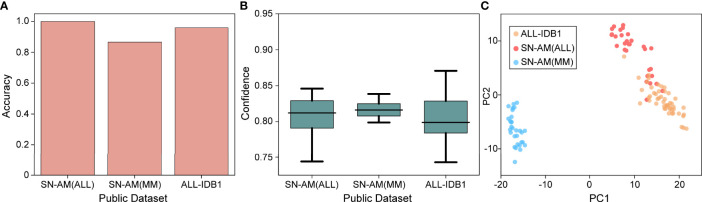
Performance on microscopy images. **(A)** Mean accuracy on the public dataset (SN-ALL, SN-MM, and ALL-IDB1) test on 40× magnifications with a single cross-validated fold. **(B)** Confidence ( ± 1 s.d.) of the prediction made by the model. **(C)** Slide-level feature space for a single cross-validated fold using PCA in the validation and test sets.

To validate the usability and generality of the proposed model for micrographs used in resource-limited areas, we also investigated the performance of our model on publicly available micrograph datasets. Three publicly available datasets, namely, ALL-IDB1, SN-AM(ALL), and SN-AM(MM), were used, including two hematological malignancies (ALL and MM). Magnifications from 30× to 100×, containing micrographs of bone marrow and peripheral blood, were used. We downsampled them to 10× magnification and tested each model using cross-validation at the same magnification. We found that the trained model performed well on micrographs, with an accuracy of 100% for SN-AM(ALL), 86.67% for SN-AM(MM), and 95.92% for ALL-IDB1 (**Figures 5A** **, B**). Furthermore, the features of different categories are still clearly separated after visualization by PCA dimensionality reduction (**Figure 5C**). We found that despite not using images from any publicly available dataset, our model also demonstrated good performance on micrographs of bone marrow smears compared to previous studies (**Table 2**). These results enhance confidence in the potential broad application of our method in the field of hematological diseases.

**Table 2:** Performance on ALL-IDB, SN-AM for different backbone studies.

Dataset	Study (Reference)	Accuracy (%)
ALL-IDB	Ahmed et al. (41)	88.25
	Palczynski et al. (42)	94.80
	Our method	95.92
SN-AM	Duggal et al. (43)	93.20
	Kumar et al. (44)	97.25
	Our method	93.67

### 3.6 Comparison With the State-of-the-Art Methods

**Figure 6:**
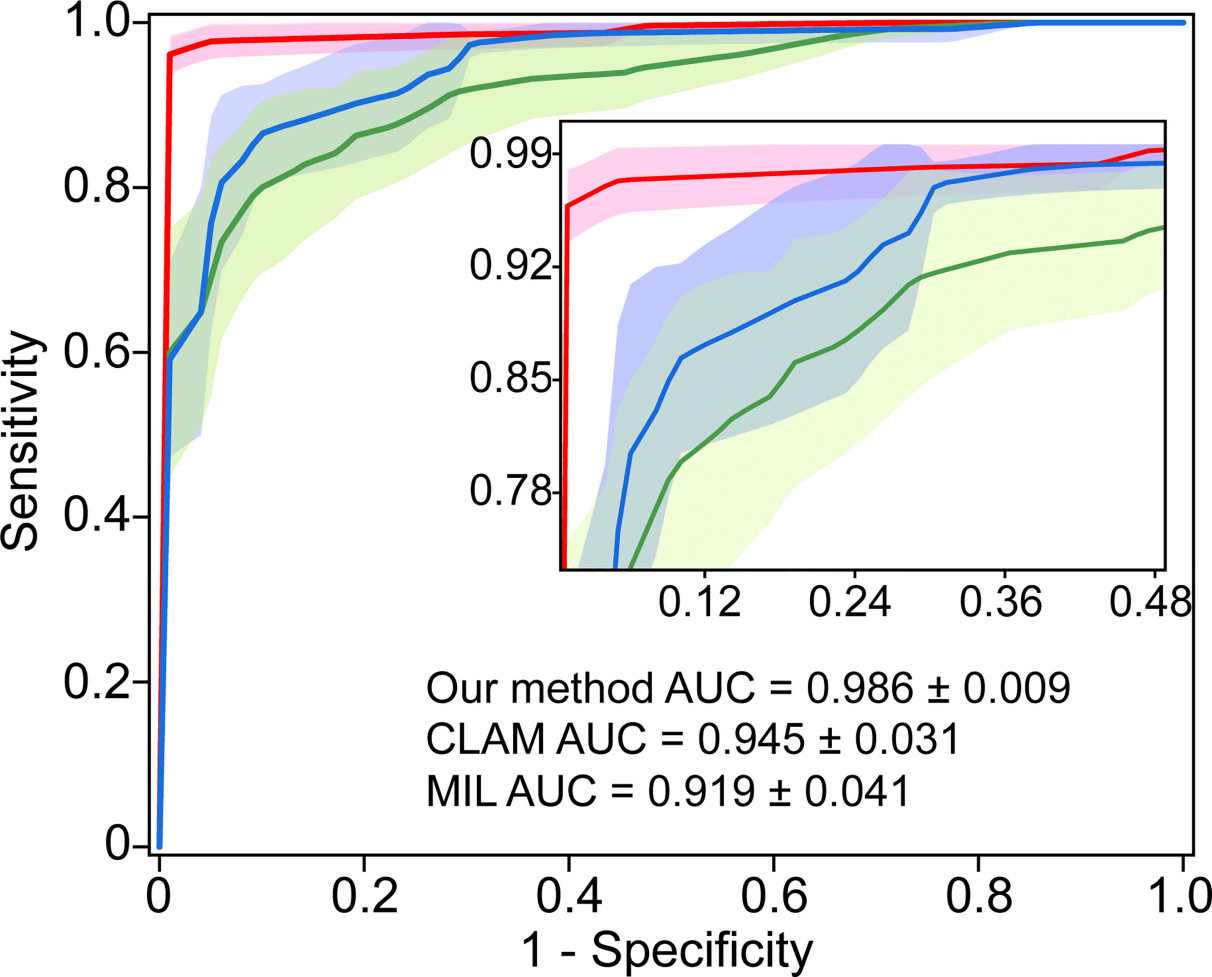
Comparison with the state-of-the-art methods. 5-fold mean test macro-averaged AUC ± s.d. of our model compared with CLAM and MIL.

We compared the performance of the proposed model with the state-of-the-art weakly supervised methods CLAM (17) and MIL (20) for WSI analysis. The CLAM and MIL were fine-tuned by changing the last output layer to 5 class to accommodate the task. The results indicated our proposed model achieved the best performance with a macro-averaged AUC ± s.d of 0.986 ± 0.009 (**Figure 6**). In addition, we found that the performance of the attention-based model (our method and CLAM) outperforms that of the max-pooling-based algorithm MIL, which indicated that the model could improve performance by assigning higher attention to regions with high diagnostic values.

## 4 Discussion

Current hematology diagnosis still relies on the manual counting of hundreds of cells on bone marrow slides due to the lack of a rapid and reliable test, which is labor intensive, time consuming, and poorly reproducible. In this study, we first developed a weakly supervised deep learning method for analyzing bone marrow smears for identifying hematologic malignancies. We found that using only slide-level labeling without detailed pixel-level or cellular annotation enables interpretable, high-performance diagnostics, which overcomes the cost of labeling and closely resembles clinical applications.

We collected a large collection of bone marrow smear WSIs and used them for training, which has more information than training a model using an expert-selected ROI (24, 26, 45) and allows for full automation. Simultaneously, we demonstrate that no adjustments are required and can be applied to the micrograph analysis of bone marrow or peripheral blood, which addresses the high imaging cost in certain resource-poor regions. The most common approach to analyzing hematological diseases using deep learning is the morphological analysis of cells in smears (27, 29, 31), which requires accurate labeling of tens of thousands of cells. Moreover, the number of different types of cells collected tends to be highly variable, leading to poor results for specific categories. We avoid the costs and inaccuracies of labeling using an attention-based network. Our results show strong performance with only slide-level tags and the ability to scale to independent test sets.

The acquisition of WSI at high magnifications is often time consuming, especially for oil lenses. Typically, a bone marrow smear takes nearly an hour at 100× magnification (approximately 22 × 45 mm^2^ slides). We analyzed the performance of using different magnification objectives in the same area and found that low magnification objectives (10× and 40×) were even better than the 100× magnification used for the cytomorphological analysis of bone marrow smears. This observation is attributed to the fact that at high magnifications, patches cropped by WSIs may only have local information on individual cells, whereas at low magnifications, there is often more information. Hence, the needed data are easy to obtain. Moreover, we first downscaled the images using a pretrained CNN, which makes the WSIs with hundreds of millions of pixels analytically efficient, leaving room for introducing other kinds of disease analysis in the future. Our method is also interpretable, generating heatmaps by introducing attention scores to visualize the significance of each area of the WSI. The results show that the regions focused on by the model are highly similar to those judged by hematologists. Thus, it may be used as an interpretable tool in applications.

Nonetheless, although our method performs well on independent test sets and publicly available microscopy image datasets, all training and test sets of WSIs were digitized by the same scanner, and the amount of available data is still limited. The performance and robustness of the model can be further validated by introducing more imaging data. The weakly supervised method we used lacks the analysis of the relationship between different positions in the same slide; rather, it treats them as independent regions. The performance may be further improved by learning the relationship between the positions of different regions. In addition, improving accuracy by introducing more information while applying it to diseases with limited data (e.g., only a few cases) and survival prediction tasks needs to be considered in future studies. In conclusion, the proposed model can be competent for the diagnosis of malignant hematological diseases, which will help to improve the realization of the fast collection of bone marrow smears and thus help to achieve fast diagnosis of hematological diseases.

## Data Availability Statement

The raw data supporting the conclusions of this article will be made available by the authors, without undue reservation.

## Ethics Statement

The studies involving human participants were reviewed and approved by Xinxiang Medical University (2019S026). Written informed consent from the participants’ legal guardian/next of kin was not required to participate in this study in accordance with the national legislation and the institutional requirements.

## Author Contributions

CZ and YY: conceived and planned the experiments. YY, CZ, and CW: designed and built the system. CW, C-XL, X-LW, Y-XN, and YW: provided and analyzed patient samples. CW and YW: processed the experimental data, performed the analysis. All authors were involved in discussing the results, writing the manuscript, and had approval of the final versions.

## Funding

This work was supported by grants from National Key R&D Program of China [2017YFA0105201]; the National Science Foundation of China [81925011, 92149304]; Key-Area Research and Development Program of Guangdong Province [2019B030335001]; The Youth Beijing Scholars Program (015), Support Project of High-level Teachers in Beijing Municipal Universities [CIT&TCD20190334]; Beijing Advanced Innovation Center for Big Data-based Precision Medicine, Capital Medical University, Beijing, China[PXM2021_014226_000026]; The Science and Technology Research Project of Henan Province [202300410323].

## Conflict of Interest

The authors declare that the research was conducted in the absence of any commercial or financial relationships that could be construed as a potential conflict of interest.

## Publisher’s Note

All claims expressed in this article are solely those of the authors and do not necessarily represent those of their affiliated organizations, or those of the publisher, the editors and the reviewers. Any product that may be evaluated in this article, or claim that may be made by its manufacturer, is not guaranteed or endorsed by the publisher.
